# Poor Appetite in Frail Older Persons—A Systematic Review

**DOI:** 10.3390/nu15132966

**Published:** 2023-06-29

**Authors:** Anna Rudzińska, Karolina Piotrowicz, Ian Perera, Barbara Gryglewska, Jerzy Gąsowski

**Affiliations:** Department of Internal Medicine and Gerontology, Jagiellonian University Medical College, University Hospital, 2 Jakubowskiego St., Building I, 5th Floor, 30-688 Kraków, Poland; anna.rudzinska@doctoral.uj.edu.pl (A.R.); karolina.piotrowicz@uj.edu.pl (K.P.); ian.perera@uj.edu.pl (I.P.); barbara.gryglewska@uj.edu.pl (B.G.)

**Keywords:** anorexia of ageing, appetite, frailty, review

## Abstract

Anorexia of aging is a common problem in older adults. Depending on the setting, its prevalence varies from about 10% (among community-dwelling older adults) to over 30% in acute wards and nursing homes. The objective of this systematic review was to establish the prevalence of poor appetite in frail persons ≥60 years of age. We performed a literature search for studies where the prevalence of anorexia of aging among frail and pre-frail old adults was reported. 957 articles on this topic were identified. After eligibility assessment, three articles were included in the review. The studies included 4657 community-dwelling older adults. The weighted total prevalence of anorexia of aging in all the included studies was 11.3%. Among frail and pre-frail participants, loss of appetite was reported in 20.5% (weighted estimate). Overall, robust status was associated with a 63% lower probability of concomitant anorexia of ageing (OR 0.37, 95%CI 0.21–0.65, *p* = 0.0005). Frailty or risk of frailty are associated with more prevalent anorexia of ageing. This has potential practical implications; however, more research, especially to elucidate the direction of the relation, is needed.

## 1. Introduction

Age-related changes in gastrointestinal motility, hormonal sensitivity and levels of pro-inflammatory cytokines make old adults especially prone to disturbances regarding energy and nutrient intake [1]. Appetite regulation is complex, and it involves many neural and hormonal mechanisms as well as psychological, economical, and social inputs [2]. Lack of appetite in older persons is called anorexia of ageing and has been described by Morley and Silver [3]. Its causes, listed by Morley and Silver, are grouped into four categories: decreased demand, decreased hedonic qualities, decreased feeding drive, increased activity of satiety factors [3]. Reduction or loss of appetite affects one in five of community-dwelling older adults. The lack of standardized tools to assess appetite and the fact that its evaluation is often based on observation of oral intake result in differences in the prevalence of appetite disturbances between studies. There is also a lack of guidelines concerning therapeutic actions when appetite disturbances have been diagnosed. The presence of anorexia of aging is associated with involuntary weight loss, lower hand-grip strength, decreased mobility, lower endurance, and in consequence decreased independence [4,5]. Physical frailty is a syndrome of physical reserve loss with advancing age that leads to increased susceptibility to stressors. There are several approaches to the diagnosis of frailty [6].

The clinical features of frailty may overlap with the clinical consequences of appetite disturbances. In 2013 it was proposed that all multimorbid persons with weight loss over 5% of their bodyweight and adults over 70 years of age should be screened for frailty [7]. 

The potential relationship between anorexia of aging and frailty can be viewed from two perspectives. The consequences of appetite disturbances that include malnutrition and nutrient deficiencies, and muscle wasting are considered to be risk factors for frailty [8,9]. On the other hand, the physical frailty phenotype is associated with fatigue, causing a deterioration in performance in activities of daily living, such as grocery shopping or preparing nourishing meals that will result in malnutrition. Likewise, social frailty, which features negative social and behavioural factors such as loneliness, lack of company during meals and poverty, may impact upon food intake and lead to anorexia of ageing and malnutrition [2].

We thus aimed to summarize the available studies concerning the prevalence of poor appetite among older persons with frailty. First, we aimed to narratively describe the published data. Further, we aimed to summarise the available numerical estimates to arrive at a pooled prevalence of anorexia of ageing. Finally, we aimed to estimate the probability of anorexia of ageing given frailty status.

## 2. Methods

### 2.1. Registration, Search Strategy and Eligibility for Inclusion

The review was performed using Preferred Reporting Items for Systematic Reviews and Meta-Analyses (PRISMA) guidelines [10]. Quality of included studies was assessed using Joanna Briggs Institute criteria [11].

We registered the review in the PROSPERO database in January 2023 (CRD 42022376585). Between 2 and 7 February 2023 we performed a search of PubMed, EMBASE and Web of Science databases using the following strategy:

- ((frail* [Title/Abstract]) AND (appetite [Title/Abstract])) OR
- ((frail* [Title/Abstract]) AND (“anorexia of ageing” [Title/Abstract])) OR
- ((frail* [Title/Abstract]) AND (anorexia [Title/Abstract])) OR
- ((frail* [Title/Abstract]) AND (CNAQ [Title/Abstract])) OR
- ((frail* [Title/Abstract]) AND (Council on Nutrition Appetite Questionnaire [Title/Abstract])) OR
- ((frail* [Title/Abstract]) AND (SNAQ [Title/Abstract])) OR
- ((frail* [Title/Abstract]) AND (Simplified Nutritional Appetite Questionnaire [Title/Abstract])) OR
- ((frail* [Title/Abstract]) AND (“loss of appetite” [Title/Abstract]))

Additionally, after performing the search and screening of the abstracts and the full texts, we screened the reference lists of the retrieved articles.

We applied the following criteria to select articles to be included in the review: (1) full reports of original research; (2) written in English; (3) human studies; (4) exploring the coexistence of appetite loss and frailty syndrome. We excluded book chapters, conference abstracts, comments, editorials, and practice guidelines.

### 2.2. Appetite and Frailty Assessment Inventories

In the included studies, appetite was assessed with Simplified Nutritional Appetite Questionnaire (SNAQ). SNAQ is a four-item questionnaire derived from the Council on Nutrition Appetite Questionnaire (CNAQ), the aim of which is to objectively evaluate appetite among older adults. A study by Wilson et al. showed that SNAQ has comparable reliability to the CNAQ scale, but as it is shorter (4 vs. 8 items), it may be more efficient in clinical settings. For SNAQ, a score of <14 points is used to identify adults with anorexia at risk of weight loss [12]. In the included studies, the following methods of frailty assessment were used: Fried physical frailty phenotype, FRAIL scale, and FRAIL-BR scale. According to Fried et al., a frailty phenotype is identified if three or more of the following symptoms are present: unintentional weight loss, self-reported exhaustion, low hand-grip strength, slow walking speed, or low physical activity [13]. The name of the FRAIL scale is an acronym derived from the words fatigue, resistance, ambulation, illness, and loss of weight, which illustrate the symptoms of frailty syndrome and constitute subsequent items of the scale [14]. FRAIL-BR is Brazilian version of the FRAIL scale used in the study by de Lima et al. [15,16].

Anorexia of aging is commonly perceived as a risk factor for frailty; however, there is a lack of prospective studies aiming to determine direction of this relationship. The objective of this systematic review was to establish the prevalence of anorexia of aging among frail persons ≥60 years of age in the cross-sectional setting.

### 2.3. Statistical Analysis

Based on the prevalence data of anorexia of ageing in frail, pre-frail and robust participants, we first calculated size-weighted prevalence for the study summary, overall and according to frailty status. Then, to arrive at a unified estimate, using the Review Manager 5 (Copenhagen, Denmark) we calculated the inverse of the variance-weighted odds ratios of presence of anorexia of ageing according to frailty status. We applied random model and considered two-sided *p* < 0.05 as the threshold of statistical significance.

## 3. Results

**Figure 1:**
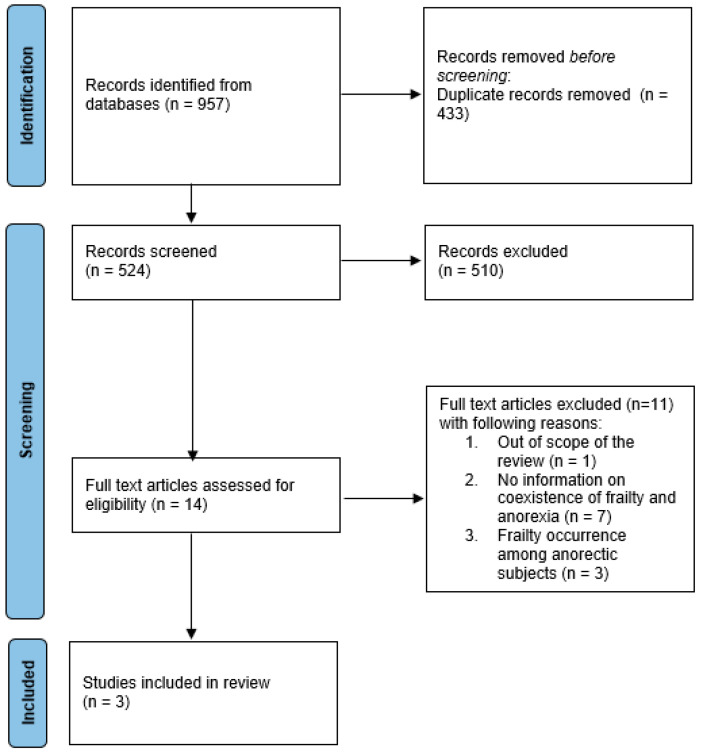
The PRISMA flowchart for the systematic review of anorexia of ageing in older adults with frailty.

We performed search of PubMed, Embase and Web of Science databases, and identified 957 items. After the removal of duplicates (*n* = 433), two independent researchers performed abstract screening (*n* = 524) and assessed the remaining full-text articles (*n* = 16) for eligibility (Figure 1). Three articles were assessed as eligible and consistent with the protocol, and consequently were included in this systematic review [16,17,18].

In Figure 1 the PRISMA flowchart that presents the process of inclusion of the studies is shown.

### 3.1. Description of Included Studies

We included three studies examining the occurrence of anorexia in frail persons ≥60 years of age, including a total of 4657 (53.15% women) participants of whom 511 (10.97%) were referred to as frail and 926 (19.88%) as pre-frail.

The study by Tsutsumimoto et al., 2017, included 4417 community-dwelling participants from the National Center for Geriatrics and Gerontology—Study of Geriatric Syndromes cohort [17]. This cross-sectional study was primarily designed to introduce a method to screen older persons for geriatric syndromes and validate preventive interventions [19]. Community-dwelling participants were monitored monthly to assess their needs for additional social support in accordance with long-term care insurance terms provided by Japan’s government. The cut-off value for anorexia of aging was set at an SNAQ of ≤13 points. Frailty was assessed using Physical Frailty Phenotype as described by Fried et al. [13]. Additionally, the cognitive status of the participants was assessed using Mini Mental State Examination (MMSE)^20^ scale and depressive symptoms were identified based on a 15-item Yesavage Geriatric Depression Scale (GDS) [20,21]. The mean (SD) age of the included participants was 75.8 (4.3) years and 52.7% of the sample were women. The mean (SD) number of medications used by the participants was 3.3 (2.8). The participants had a mean (SD) MMSE score of 26.2 (2.3) and a mean (SD) GDS score of 2.8 (2.7). Frail (*n* = 472) and pre-frail (*n* = 853) patients were older (*p* = 0.001), experienced appetite loss more often (*p* = 0.001) and had worse self-reported health (*p* = 0.001) than the robust participants (Table 1). The total prevalence of anorexia of aging in the study participants was 10.6% (non-frail: 7.9%, pre-frail 14.8%, frail 21.3%).

In the logistic regression models adjusted for important confounders that included age, gender, BMI, and number of medications, anorexia of ageing was associated with greater risk of concomitant frailty (OR 1.86, 95% CI 1.39–2.49, *p* = 0.001). In similar analysis, anorexia of ageing was also associated with increased risk of coexisting pre-frailty (OR 1.59, 95% CI 1.25–2.02, *p* = 0.001). These results point towards a linear increase in the risk of concomitant appetite disturbances with an increasing load of frailty phenotype. No significant differences between body mass index (BMI) scores were observed according to frailty status. Notably, weight loss, one of the components of frailty, has been associated with occurrence of anorexia of aging (OR 1.37, 95% CI 1.05–1.79, *p* = 0.019).

The cross-sectional study by Alex et al. included 134 community-dwelling participants from Malaysia, who responded to a survey about occurrence of geriatric syndromes using an online tool [18]. The primary objective of the study was to check utility and validity of that form of screening. The SNAQ cut-off value for anorexia of aging was ≤14. The FRAIL scale was used to screen for frailty among participants. The mean (SD) age of the participants was 66.4 (5.3) years; 64.9% of the study group were women. Of the survey’s participants, 27.8% were reported to have cognitive impairment as assessed with Alzheimer’s Dementia Screening Interview (AD8) [22]. Frail (*n* = 6) and pre-frail (41) groups were analysed together and compared with the non-frail (*n* = 87) participants. The total prevalence of anorexia of aging in the study was 27.2% (24.1% in robust participants and 48.9% in the pre-frail and frail participants). Frail and pre-frail persons more often experienced appetite loss (*p* = 0.007) and had worse self-reported health measured as a single item (yes/no, *p* = 0.001) (Table 1).

The study by De Lima et al. was a cross-sectional analysis including data of 106 geriatric outpatients from the Multimorbidity and Mental health Cohort Study in Frailty and Aging (MiMiCS-FRAIL) that aimed to assess associations between frailty, depression and multimorbidity [16,23]. The study took place in Brazil. Participants were recruited by the general practitioners, specialists and a ‘university screening outpatient clinic’ [16]. The SNAQ cut-off value for appetite loss was <14 points. FRAIL-BR was used for frailty assessment. The mean (SD) age of the participants was 71.4 (8.0) years, and 58.5% of the group were women. The prevalence of anorexia of aging was 19.8% (2.4% among non-frail participants, 28.1% among pre-frail participants, 33.3% in frail participants). Frail (*n* = 33) and pre-frail (*n* = 32) participants were significantly older than non-frail (*n* = 41) ones (*p* = 0.002). In both unadjusted models and after adjustment for SARC-F, age and abdominal circumference, appetite was significantly associated with the risk of concomitant frailty (adjusted OR 0.68, 95% CI 0.50–0.92; *p* = 0.012 for pre-frail, and OR 0.64 95%CI 0.45–0.90; *p* = 0.009 for frail).

### 3.2. Anorexia of Ageing–Prevalence and Probability of Co-Exitence with Frailty

The weighted total prevalence of anorexia of aging in all the included studies was 11.3%. Among frail and pre-frail participants, loss of appetite was reported in 20.5% (weighted estimate).

**Figure 2:**
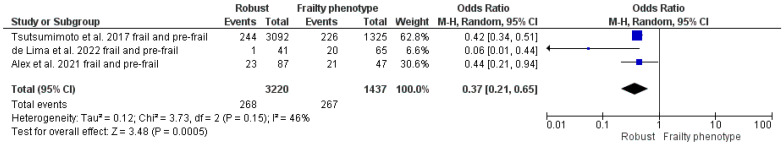
Odds ratios of anorexia of ageing in robust versus frail and pre-frail older persons. Pooled analysis of cross-sectional data from three included studies [16,17,18].

We then calculated the odds ratios of anorexia of ageing associated with robust versus frailty phenotype status. Overall, the robust status was associated with 63% lower probability of concomitant anorexia of ageing (OR 0.37, 95%CI 0.21–0.65, *p* = 0.0005, I-square 46%) (Figure 2).

## 4. Discussion

In the three reviewed studies that in total included 4657 participants, we found that appetite loss was present in 11.3% of participants [16,17,18]. Overall, the studies found a relation between anorexia of ageing and pre-frailty or frailty. However, the studies were heterogenous as to the population included (two face-to-face assessment in general population, one an online survey), the methodology to assess frailty (different tools and cut-offs) and anorexia of ageing (different cut-offs). Overall, based on the Briggs criteria, the studies were of moderate quality (Table 2) [24].

Research on appetite disturbances among old adults has been conducted since 1985, when Morley used the term ‘anorexia of aging’ for the first time [25]. However, only a few studies included the assessment of both anorexia of ageing and physical frailty.

The first study that aimed to provide data on both anorexia of aging and frailty was the ilSIRENTE study, conducted in 2004 in persons aged 80 and over. However, the lack of detailed information concerning the methodology of frailty assessment rendered the study ineligible for the inclusion in the review. It was assessed that we could not include this study in the present review. The results of this study indicated that subjects with anorexia (ascertained with two simple single-item questions) have worse physical performance and muscle strength than persons with normal appetite. At the same time, in this study, there were no significant differences between body mass index of persons with anorexia and with preserved appetite [4]. This observation is consistent with results of the study by Tsutsumimoto et al., 2017, included in this review [17].

We also identified two reports (Tsutsumimoto et al., 2018, Zukeran et al., 2022) that were based on data from the abovementioned studies, and that were focused on the prevalence of frailty among the anorexic subjects [26,27]. As the articles expand on the information in the initial reports, it is important to discuss their results in the context of the present review. In both studies, subjects with poor appetite were more often recognized as frail or pre-frail than persons with preserved appetite. Importantly, the mentioned studies used data from larger datasets that were used also in studies included in this review. The report by Tsutsumimoto et al. (2018) included 4393 participants from the abovementioned National Center for Geriatrics and Gerontology—Study of Geriatric Syndromes cohort. The average (SD) age of the included participants was 75.9 (4.3) years. Persons who experienced appetite disturbances were older (*p* < 0.001), had lower BMI (*p* < 0.001) and more often were identified as frail and pre-frail (*p* < 0.001) according to Fried criteria than persons without anorexia of aging. In the logistic regression models adjusted for age, sex, education, BMI, polypharmacy, hypertension, diabetes mellitus, total serum protein, serum albumin, living alone vs. with someone, alcohol consumption, smoking, depressive symptom, decline of physical function, and cognitive decline, pre-frailty (vs. robustness) was associated with greater probability of concomitant anorexia of ageing (OR 1.53, 95%CI 1.16–2.32, *p* = 0.007). In a similar analysis, frailty (vs. robustness) was also associated with a greater probability of concomitant anorexia of ageing (OR 2.44, 95%CI 2.00–4.59, *p* < 0.001).

Zukeran et al. analysed data of 122 participants (58.2% women) from the MiMiCS-FRAIL cohort. Frailty status was assessed using the Frailty Index (FI-36). The cutoff for anorexia of aging was ≤14 points. The mean age of the participants was 71.7 (7.9) years. Participants with anorexia of ageing were older (*p* = 0.01), had higher FI-36 (*p* = 0.014) and GDS results (*p* < 0.001) than persons without appetite loss. In unadjusted analysis the Frailty Index 36 greater by 1 score was associated with 6300% greater probability of concomitant anorexia of ageing (*p* = 0.04) however the model may have been inflated, possibly by outliers. However, in a logistic regression model adjusted for gender, age, ethnicity, schooling, and GDS, a score on the Frailty Index 36 greater by 1 was not associated with greater probability of concomitant anorexia of ageing (OR 2.07, 95% CI 0.42–103.21, *p* = 0.71).

The results of studies concerning anorexia of ageing in frail or pre-frail persons should be interpreted taking into consideration the multidimensional aetiology of appetite disturbances among old persons. The Longitudinal Ageing Study Amsterdam APPETITE project by Scheufele et al. investigated anorexia of ageing and its correlates in a sample from general population [28]. Poor appetite was assessed using the second item of the Centre for Epidemiologic Studies Depression Scale (CES-D) [29]. Frailty was not assessed in this study, but functional limitations, multimorbidity and polypharmacy, physical activity, and loss of weight were addressed. Poor appetite was identified in 15.6% of study participants. Of the factors under study, polypharmacy, and weight loss in the previous six months, but not multimorbidity, were related with appetite loss. BMI itself was not associated with the presence of anorexia of ageing and neither was the level of physical activity. Other factors associated with poor appetite were chewing impairment, anxiety, and presence of depressive symptoms, poor self-perceived health, and female sex.

According to results by van der Meij et al., the presence of senile anorexia in community setting was reported for 21.8% old adults taking part in the study [30]. According to Donini et al., appetite disturbances were more frequently observed among women (in hospital and nursing homes setting), older adults and in hospital settings [31]. In a community setting, anorexia of ageing was more often recognized among men than women, 11.3% and 3.3%, respectively. In nursing homes, anorexia of ageing was present among 27.2% of men and 34.1% of women. The prevalence of anorexia in the rehabilitation or acute wards was 26.7% and 33.3% for men and women, respectively [31]. In a study by Pilgrim et al., among hospitalized, older female patients, an even higher rate of anorexia of ageing reaching 42% was observed [32]. In the ICFSR Task Force Report on Appetite Loss and Anorexia of Aging in Clinical Care, depression, neurological, gastrointestinal, and inflammatory diseases were highlighted as anorexia determinants among old patients [33]. The IlSIRENTE study showed also a significant correlation between the presence of diseases such as COPD and congestive heart failure, as well as the total number of illnesses and appetite disturbances [4]. The same ICFSR Report considers the role of appetite in physical frailty development putting emphasis on the fact that most of the tools to identify frailty contain items for the assessment of nutritional status [33]. Appetite status, just like the frailty phenotype, is influenced, in a reciprocal manner, by many physiological, pathological, and social factors, resulting in malnutrition, weight loss, and aggravation of frailty.

As so-called geriatric giants tend to overlap one another, there is a need to consider anorexia of aging in the context of problems such as sarcopenia and disability, dementia, and depression [33]. The results of the aforementioned ilSIRENTE study indicate that subjects with anorexia of aging have worse ADL and IADL status, worse performance in the 4 m walking test and lower hand grip strength than persons with preserved appetite [4]. No significant differences were found between appetite status and body mass index of the participants. Those with anorexia of ageing were also shown to have worse cognitive performance as measured with the Minimum Data Set—Home Care (MDS-HC) tool [34]. In general, old adults diagnosed with dementia are at a high risk for development of malnutrition. The European Society for Clinical Nutrition and Metabolism (ESPEN) guidelines recognize that certain determinants of poor appetite, such as smell and taste disturbances, are common among patients with dementia, especially at the preclinical stage [35].

Currently, there are no agreed guidelines regarding the evaluation of appetite that would include validated biomarkers. Consequently, researchers and clinicians usually use the following methods to recognize anorexia of aging: asking plain-language questions about the desire to eat, using the second item of the CES-D questionnaire concerning appetite loss, using Simplified Nutrition Appetite Questionnaire or its full version, the Council on Nutrition Appetite Questionnaire, and observation of oral intake. However, these methods do not fully address the problem of appetite loss, especially in the populations especially prone to its disturbances such as persons with dementia or depression.

ICFSR report proposes that persons with severe cognitive impairment should be excluded from the future drug trials on anorexia of aging [33]. At the same time, due to the high prevalence of disordered eating patterns among persons with dementia and difficulty with communicating their needs for dietary adjustments, this group is at a particular risk of developing malnutrition. As for dementia and disordered eating patterns, existing scales such as the Edinburgh Feeding Evaluation in Dementia Scale indicate the need for further investigation of appetite among those with cognitive impairment in studies with cross-sectional methodology and studies involving caregivers of patients with dementia [36].

A changed appetite is one of the most common depressive symptoms. When in depressed mood, adults aged ≥ 60 years probably more often experience appetite loss than increase [37]. The consequences of decreased appetite during depression may include weight loss, nutrient deficiencies, and poor nutritional status. These constitute increased risk for development of cognitive frailty [38,39]. The link between loss of appetite and depression is probably reciprocal, as lower appetite and nutritional intake may be a symptom of depression, but also an individual experiencing appetite loss may develop depression as a consequence of malnutrition or as a coexisting condition [39]. Consequently, screening for appetite disturbances in depressed old patients followed by a tailored nutritional intervention could prevent weight loss and development of malnutrition. At the same time, screening for depressive symptoms should be considered whenever patient reports loss of appetite, even without weight loss or with coexisting obesity.

Varied tools have been developed to assess frailty. The screening is often performed with the FRAIL scale [15]. The ICFSR task force recommends using the following tools for frailty screening: Rockwood’s Clinical Frailty Scale (CFS) [40], the International Association of Nutrition and Ageing FRAIL scale, and Edmonton Frailty Scale (EFS) [41,42]. As there is no consensus on which method is best to use, comparison between studies aiming to assess frailty is sometimes impossible.

The quality of the studies included in this review was moderate according to Joanna Briggs Institute Criteria.

Our review showed that there are not enough data regarding prevalence of anorexia of aging among frail and pre-frail persons. There is also insufficient evidence regarding the diagnosis and treatment of anorexia of aging. In 2022 The International Conference on Frailty and Sarcopenia Research (ICFSR) Task Force published their summary of existing evidence and recommendations on the topic of anorexia of aging, partially addressing the issue of the interplay between appetite loss and frailty. As stated in that report, the development of both anorexia of aging and frailty syndrome involve complex mechanisms of physiological and pathological factors, including hormonal, psychological, and social determinants. The report points out that more research is needed on the topic of appetite loss and frailty [33].

The results of previous studies indicate that people with frailty syndrome are more likely to experience appetite disorders [16,17,18]. However, there is a lack of prospective studies that could confirm the direction of this relationship and address the issue of causality. Likewise, there is a lack of intervention studies devoted to the possibility of reversing appetite disorders and the impact of appetite improvement on the patient’s functional status. According to previous research on the interventions to restore appetite, none of the studies examined outcomes related to frailty syndrome itself. In the studies included in the review by Cox et al. concerning possible interventions to increase appetite, frailty-directed interventions were not included [43].

In studies devoted to the assessment and treatment of appetite disorders, there is also a noticeable lack of clarity on the terms used to describe appetite disturbances and distinguish them from malnutrition, which may or may not be present with anorexia of aging. It may be due to the fact that anorexia of aging is a relatively newly recognized phenomenon, and there are no guidelines for anorexia of aging assessment and treatment that would help determine the tools used to evaluate appetite and the vocabulary used to describe its disorders.

As research on frailty syndrome grows, researchers indicate that frailty, understood as reserve loss in the physical domain, is often accompanied by cognitive impairment and reduced social resources due to shrinking of social networks, constituting constructs of, respectively, cognitive frailty and social frailty. As both social and cognitive determinants are considered important for maintaining proper appetite and nutritional status, this implies the need to conduct further research on appetite and frailty, considering areas that are often impaired in the course of aging.

Limitations and strengths. Our review must be considered within the context of its limitations. First, only few studies were identified that finally met the predefined inclusion criteria. Second, after the assessment according to the Briggs criteria, the quality of those studies was moderate at best. Third, the published data allowed only limited meta-analysis to be performed, and we were not able to contrast robust with pre-frail and frail persons separately. Instead, we calculated the odds ratio of anorexia of ageing in robust versus pre-frail and frail persons combined (frailty phenotype). However, based on the review, we could pinpoint the important knowledge gaps and chart the areas in need of more stringent study. Even though several of the studies that we at first identified as potentially includable did not meet the entry criteria in the end, we took care not to exclude them from the discussion. In a field so much lacking in well-standardized research as anorexia of ageing, it is important to have the fullest possible view of the research studies that have been performed.

In conclusion, anorexia of ageing is an important health-related issue in older individuals. This is especially true of older persons with frailty or pre-frailty. Identification of patients with anorexia of ageing is essential to design dietary interventions likely to improve both the physiologic status and the functionality of these patients. However, more high-quality research is needed both to assess the causative relations between frailty and anorexia of ageing, and to present reliable interventions to tackle the problem of anorexia of ageing.

## Figures and Tables

**Table 1:** The characteristics of the studies included in the review.

	Author, Publication Year (Reference)	Cohort				Frailty		Appetite according to Frailty Status			
		*n*	Country	Setting	Age * <minimum, (Mean [SD]) Years	Tool (Reference)	% (*n*)	Tool	Anorexia of Ageing (%) within Frailty Status Group (*n*)	*p*	SNAQ Mean (SD)
1.	Tsutsumimoto et al., 2017 [17]	4417	Japan	Community	≥70 (75.8 [4.3])	Fried et al. [13]	FRAIL 10.7% (472)	Simplified nutritional appetite questionnaire (SNAQ); cut-off: ≤13	Among frail participants: 21.2% (100)	<0.001 (ANOVA)	14.5 (1.5)
							PRE-FRAIL 19.3% (853)		Among pre-frail participants: 14.8% (126)		15.0 (1.5)
							NON-FRAIL 70% (3092)		Among non-frail participants: 7.9% (244)		15.5 (1.4)
2.	Alex et al., 2021 [18]	134	Malaysia	Online survey	≥60 (66.4 [5.3])	FRAIL [14]	FRAIL: 4.5% (6)	Simplified nutritional appetite questionnaire (SNAQ); cut-off: ≤14	Among frail and pre-frail participants: 24.1% (21)	0.007	…
							PRE-FRAIL: 30.6% (41)				…
							NON-FRAIL: 64.9% (87)		Among non-frail participants: 48.9% (23)		…
3.	de Lima et al., 2022 [16]	106	Brazil	Community	≥60 (71.4 [8.0])	FRAIL-BR [15]	FRAIL: 31.1% (33)	Simplified nutritional appetite questionnaire (SNAQ); cut-off: ≤14	Among frail participants: 33.3% (11)	0.005 (ANOVA)	…
							PRE-FRAIL: 30.2% (32)		Among pre-frail participants: 28.1% (9)		…
							NON-FRAIL: 38.7% (41)		Among non-frail participants: 2.4% (1)		…

* No information concerning the upper age limit was included; ellipsis denotes lack of information.

**Table 2:** Quality of included studies according to Joanna Briggs Institute criteria.

	Tsutsumimoto et al., 2017 [17]	Alex et al., 2021 [18]	De Lima et al., 2022 [16]
Was the sample frame appropriate to address the target population?	Yes	Yes	Yes
Were study participants sampled in an appropriate way?	Unclear	No	No
Was the sample size adequate?	Unclear	Unclear	Unclear
Were the study subjects and the setting described in detail?	Yes	Yes	Yes
Was the data analysis conducted with sufficient coverage of the identified sample?	Yes	Yes	Yes
Were valid methods used for the identification of the condition?	Yes	Yes	Yes
Was the condition measured in a standard, reliable way for all participants?	Unclear	Yes	Yes
Was there appropriate statistical analysis?	Yes	Yes	Yes
Was the response rate adequate, and if not, was the low response rate managed appropriately?	Unclear	Yes	Yes
Overall quality	Moderate	Moderate	Moderate

## Data Availability

Not applicable.

## PROSPERO registration

(CRD 42022376585).
